# Event and Comment

**Published:** 1906-09-15

**Authors:** 


THE
### DENTAL REGISTER.

Vol. LX. September 15, 1906. No. 9.

### EVENT AND COMMENT.

### Thymophene.

In the July 1906, *Dental Cosmos*, Dr. Kirk recomme⁻ds a mixture of Thymol and Phenol in equal parts, which he names "Thymophene," as an excellent disinfectant canal dressing. Dr. Herman Prinz, in the August DENTAL ERA, cites the fact that he recommended the same preparation for the same purposes substantially in an article printed in the DENTAL REGISTER, September, 1900. In referring to our files we find that Dr. Prinz is substantially correct. This is another case of independent discovery by different men of substantially the same thing. There can of course be no criticisms laid against Dr. Kirk for publishing the matter as original as it certainly was so far as he is concerned, It is a perfectly natural outcome of the use of these remedies independently and combined by our profession as they have been to a large extent for the past ten years. The profession is to be congratulated in having two such able practitioners and scientific thinkers evolve a remedial agent that they can so heartily commend. It isn't so much to the glory of Prinz, or Kirk, that this has transpired as it is to the benefit of suffering mankind. Therefore we are most grateful to both, Drs. Prinz and Kirk for calling our attention to this good remedy which we trust shall prove its right to the character given it by such worthy sponsors.

**Thymocamphene.**

In commenting on the discovery of Thymophene by Dr Kirk, Dr. Prinz says that he finds the addition of camphor gum to the Thymol and phenol valuable in still further reducing the caustic and irritating properties of these drugs. He adds the camphor in the following proportions: to two drams each of thymol and phenol one dram of camphor gum, mixed in a dry test tube and fused with a low heat. The camphor thoroughly dissolves and the result is a stable liquid at ordinary temperatures. As all of the drugs used are volatile a fragrant but not unpleasant odor results. If as suggested by Dr. Kirk, the thymol and phenol combination should be called Thymophene. We would suggest that the camphor preparation might well be called Thymocamphene.

How much it will mean to the dental profession when practitioners shall use only remedies of known elements and potencies, rather than the secret formulas of the commercial agent. In this day of science there should be no occasion for the prevailing habit of treating dental diseases with nostrums of unknown composition or worth, especially when we have so many valuable remedies that are unpatented, and can be vouched for by strictly reliable therapeutists. The extensive use of the secret nostrum is not only ethically and legally wrong, but it tends to pauperize the professional intellect to an extent that is not easily comprehended by the unthinking users of the so called ''sure cures'' which are guaranteed to never fail and to be perfectly harmless to the patient. While we do not altoghter commend the idea of formulating all our remedial agents, as might seem to be intimated in this comment, we are not sure but the best means the profession can employ to drive out of existence the secret remedies is to replace them with known ones that have been scientifically and clinically tested.

**What We Seem and What We Are.**

In another part of this issue we reprint an editorial from one of our esteemed contemporaries on what the author pleases to call ''A Few Gentle Thumps.'' He aims to set forth the real standing of the dentist and gives what he believes to be the reasons for this status. For instance he says, "that if any dentist gets puffed up with his own importance and imagins that either he or his profession is equal to the physician and the medical profession, then this Mr. Dentist, deserves a few good bumps for becoming so chesty." If the author means to comment on some individual member of our profession, whom he may know to hold such egotistical vanities as he enumerates, for purely personal reasons for preferment, we of course can take no exceptions to his remarks; but in the next paragraph, he says; "dentistry, as followed by nine-tenths of its practitioners, is a narrow and narrowing profession." Here we would take exceptions, and want to say, that we believe more than one-tenth of our practitioners are spending their lives and energies with just as broad and liberal a view of our useful calling as in any other profession. It would not be difficult to cite, convincing instances of heroic and broad minded devotion to our art, and of an equally broad sympathy with many of the noblest occupations that engage the sympathy and support of great and liberal men everywhere. Where for instance could you find a man of broader culture and more active sympathy with every important religious or political event of his day, than the late Doctor Jonathan Taft? But we do not need to cite such illustrious examples. In almost every town of sufficient consequence to support two or more dentists you may find, perhaps, a most noble character in the person of a quiet unheard of dentist, who is doing his work with no noisy fanfare, but none the less faithfully and effectively. And just to the extent that he is fulfilling the necessity for his existance is he great and broad and useful. Who knows or cares anything about the people who built the wonderful temple of Diana, or the equally wonderful Egyptian Pyramids, or any other great works of the past? None of these people can compare in nobility of character or true greatness with the lowly Nazarene Carpenter, who simply did good and taught people to love and serve each other.

It is true some callings give larger opportunities for popular recognition than others and it may be that physicians have more vital responsibilities to deal with than the dentist, and yet he may not save more lives than the policeman or the member of the life saving crew. No one has a right to belittle his calling, because it is an inconspicious one, so long as it is helpful and essential to the welfare and happiness of mankind. And if it were possible that only one man in the entire profession of dentistry had earned the right to be called a broad minded and cultured citizen, this would render it not only possible, but essential that all members of the profession should claim the right to be classed as cultured gentlemen, and should strive with all their powers to earn the title which belongs to them; not for the glory which it might bring, but for the happiness which comes as a result of feeling that one has a place in the great world's work and that he is filling it. This is the dentists Heaven just as much as it is the physicians' or the clergyman's.

